# Telecom single-photon emitters in GaN operating at room temperature: embedment into bullseye antennas

**DOI:** 10.1515/nanoph-2022-0659

**Published:** 2023-02-20

**Authors:** Max Meunier, John J. H. Eng, Zhao Mu, Sebastien Chenot, Virginie Brändli, Philippe de Mierry, Weibo Gao, Jesús Zúñiga-Pérez

**Affiliations:** Université Côte d’Azur, Centre National de la Recherche Scientifique (CNRS), Centre de Recherche sur l’Hétéro Epitaxie et ses Applications (CRHEA), Rue Bernard Gregory, 06560 Valbonne, France; Division of Physics and Applied Physics, School of Physical and Mathematical Sciences, Nanyang Technological University, SPMS-PAP-03-06, 21 Nanyang Link 637371, Singapore; A*STAR, (Agency for Science, Technology and Research), Institute of Materials Research and Engineering, 2 Fusionopolis Way 138634, North Tower, Singapore; Majulab International Joint Research Unit UMI 3654, CNRS, Université Côte d'Azûr, Sorbonne Université, National University of Singapore, Nanyang Technological University, Singapore; Division of Physics and Applied Physics, School of Physical and Mathematical Sciences, Nanyang Technological University, SPMS-PAP-03-06, 21 Nanyang Link 637371, Singapore;and Center for Quantum Technologies, National University of Singapore, Singapore 117543, Singapore

**Keywords:** bullseye antennas, GaN, purity, room-temperature, single-photon emitter, telecom

## Abstract

The ideal single-photon source displaying high brightness and purity, emission on-demand, mature integration, practical communication wavelength (i.e., in the telecom range), and operating at room temperature does not exist yet. In 2018, a new single-photon source was discovered in gallium nitride (GaN) showing high potential thanks to its telecom wavelength emission, record-high brightness, good purity, and operation at room temperature. Despite all these assets, its coupling to photonic structures has not been achieved so far. In this article, we make a first step in this direction. First, we analyze whether stacking faults are indeed a necessary condition for obtaining such emitters in GaN layers. Then, we discuss the challenges associated to a low spatial density and to a spectrally wide distribution of emitters, which necessitate their location to be determined beforehand and the photonic structure resonance to be tuned to their emission wavelength. The design and fabrication of bullseye antennas are thoroughly described. Finally, we fabricate such bullseyes around telecom emitters and demonstrate that the embedded emitters are able to sustain the necessary clean-room process and still operate as single-photon emitters after the fabrication steps, with room-temperature purities up to 99% combined with repetition rates in the order of hundreds of kHz. The findings in this work demonstrate that telecom single-photon emitters in GaN operating at room temperature are well adapted for single-photon applications where brightness and purity are the required figures of merit, but highlight the numerous difficulties that still need to be overcome before they can be exploited in actual quantum photonic applications.

## 1 Introduction

The first true single-photon source (SPS) was used in 1974 for blue-sky research as an experimental proof of the quantum nature of light through photoelectric effect, and was based on cascade transition of calcium atoms [1]. Since then, in the context of the so-called second quantum revolution and in response to various needs and applications [2, 3], other types of such source emerged, based for example on single atoms [4], nonlinear processes [5], or atom-like systems embedded into semiconductors [6]. Nevertheless, no perfect SPS has yet been discovered, and each of these sources responds to specific figures of merit necessary to some applications only.

In the case of quantum communications, and in particular the encryption of information through quantum channels, commonly gathered under the term “quantum key distribution” (QKD) [7], two properties are required to get closer to the ideal source: (1) the single-photons must lie in the telecom band to benefit from the lowest transmission loss when propagating through optical fibers and (2) the source must operate at room temperature for practical scaling and integrability [8, 9]. For example, of all the kinds of semiconductor quantum dots [10], yet broadly understood, studied, and even commercialized (Quandela), none fulfills both these criteria [11].

On the other side, only special cases of atomic cascade sources [12] and parametric down conversion [13], which is the most commonly used today due to its ease of implementation, will present simultaneously the two characteristics mentioned above. However, due to their intrinsically low brightness and the difficulty to scale them up or integrate them with other devices, solid-state SPS gained interest over the last decades. One can cite defects in silicon carbide (SiC) [14, 15], carbon nanotubes [16], transition metal ions (TMI), or rare-earth ions (REI) embedded into semiconductors. Their development and differentiation rely in the level of physics understanding and in the engineering achievements to enhance properties such as purity or brightness.

Alongside, a new type of SPS also following these criteria was discovered in 2018, based this time on GaN [17]. It immediately appeared as a particularly promising candidate, due to its record-high brightness at the time, its competitive purity, and its large polarizability. Moreover, the host material being GaN, it could also benefit from all the developments associated to the solid-state lighting technology for blue and white GaN-based LEDs [18].

Surprisingly, no further report has been published since that first paper in 2018, although several groups have continued to work on visible SPS in GaN [19, 20]. The logical route for the development of a new SPS usually involves the understanding of its origin, which enables its deterministic generation and facilitates its technological development, like the embedding into photonic structures. As none of these steps has been pursued yet, we propose in this article to move forward and assess the first integration of such room-temperature GaN-based emitters operating in the near-IR and telecom wavelength ranges with photonic structures. After studying a potential origin for these emitters and highlighting their wide spectral distribution, we expose a suitable simulation and processing scheme for their coupling to bullseye antennas. We then demonstrate the feasibility of such integration, in particular the compatibility of the SPS with the necessary processing of such structures. Finally, we characterize the emitters after the fabrication and assess properties bringing new challenges ahead.

## 2 Generation of telecom SPS in GaN

A practical SPS should benefit from sufficient investigation and technological background to allow for its efficient fabrication and integration. The current knowledge on telecom GaN emitters only gathers their basic optical properties such as zero-phonon line (ZPL), count rate, lifetime, and purity. In particular, it has not yet been studied whether all crystalline GaN samples contain such emitters or even if, within one such type of sample, only specific growth/deposition conditions result in their generation. In this section, we first study the impact of certain growth parameters on the presence of telecom SPS in GaN.

One interesting property of these sources, raised already in reference [17], is the wide distribution of ZPL (ranging almost continuously from 1.05 to 1.35 μm), as well as the wide linewidth distribution, with SPSs displaying linewidths from some nanometers to some tens of nanometers. This behavior is relatively unusual for solid-state color centers and suggested either the existence of several point defects differing in nature, or of several combinations of point/structural defects, as in the more thoroughly studied case of SiC [21, 22]. In line with this latter hypothesis, Zhou et al. [17] had suggested that the combination of a point defect and a stacking fault (SF) could explain the shift in ZPL.

A SF is an extended planar defect where one or several layers of the material break the stacking order. In the case of wurtzite GaN, with an ABABAB … ordering, the SF appears as one (or several) cubic ABC segment surrounded by usual hexagonal ABABABAB structures [23]. Two kind of SFs can coexist in GaN: basal stacking faults (BSF) and prismatic stacking faults (PSF), depending whether the defect is contained within the c-plane or inclined to it. As indicated earlier, BSFs in GaN can be regarded as cubic (zincblende ZB) inclusions within the usual hexagonal (wurtzite WZ) lattice of the GaN. The difference in band gap between these two crystallographic structures, with ZB GaN displaying a smaller bandgap than hexagonal GaN, leads to the creation of a cubic GaN quantum well within WZ GaN quantum barriers. Interestingly, due to the electrostatic polarization mismatch between the polarless cubic insertion and the polar WZ GaN [24], a larger thickness of the SF will lead, through the quantum confined Stark effect, to a red-shift of the emission wavelength as well as a larger separation between the electrons and holes wavefunctions. The hypothesis of [17] is that a point defect in the vicinity of such extended defect would lead, by variation of the geometry of the complex as well as the type of the involved SF, to a shift in emission wavelength.

**Figure 1:**
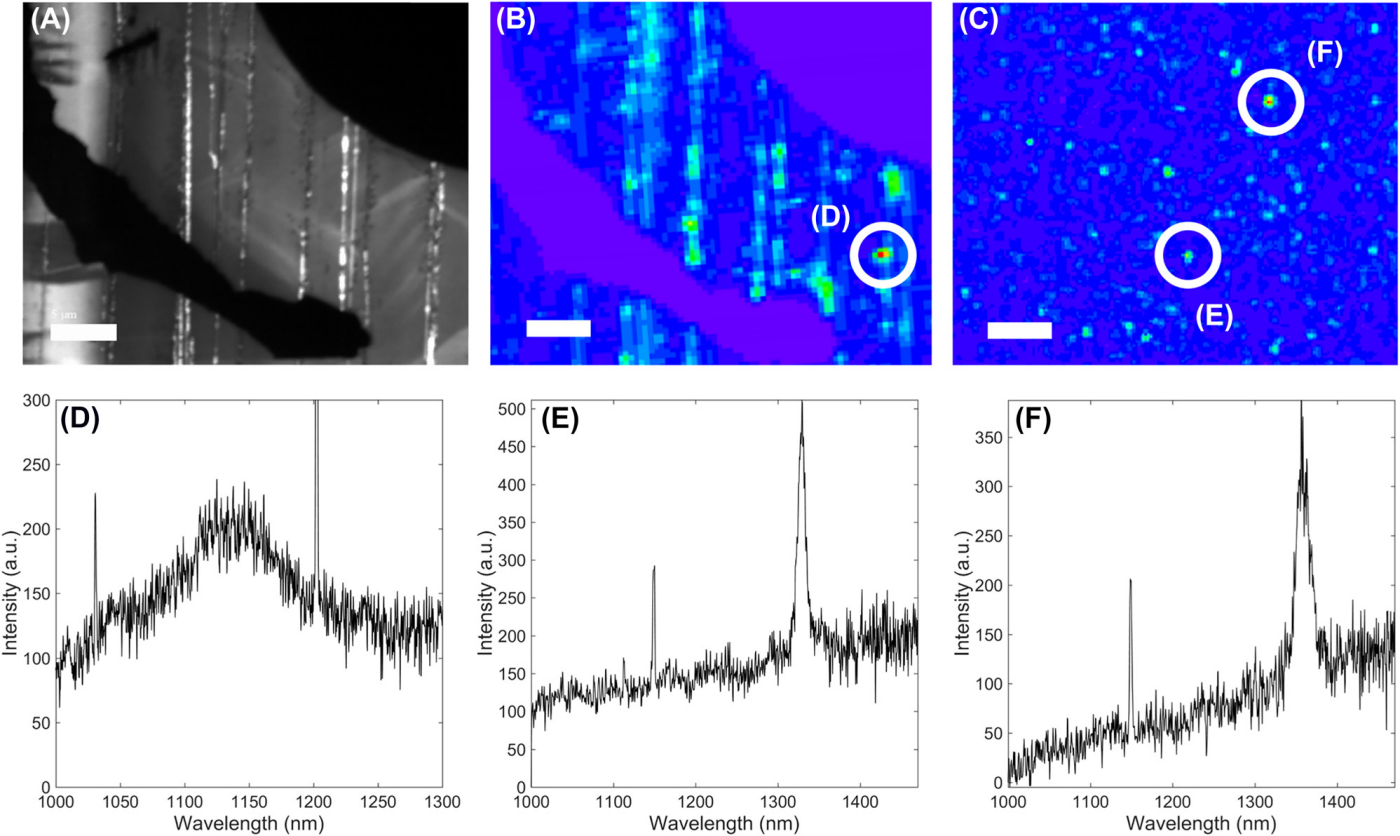
Stacking faults in GaN and telecom single-photon emitters: (A) Cathodoluminescence image taken at 77 K of a semi-polar GaN on a patterned sapphire substrate with metallic alignment marks. The marks appear as black areas, while the stacking faults luminescence stands out as bright lines. (B) Room-temperature micro-photoluminescence image of the same sample at the same spot. Defects at the vicinity of stacking are visible by higher luminescence spots. (C) Room-temperature micro-photoluminescence image of a polar GaN on sapphire sample. Scale bars represent 5 μm. White circles are placed around some emitters whose photoluminescence spectra at room-temperature are shown in (D), (E), and (F).

To verify this hypothesis, we aimed at creating SF in localized regions and study the presence of SPS in their surroundings. For that we used a semipolar (11 $\bar{2}$ 2) GaN grown on a patterned sapphire substrate (PSS), similar to the samples of reference [25]. It allows not only the creation of BSF at deterministic positions thanks to the PSS but also to follow their propagation all the way to the surface due to the tilt of the polar axis. After processing alignment marks, we could, therefore, locate the BSF with respect to the mask, by cathodoluminescence (CL) measurements at low temperature, as shown by the bright lines in Figure 1A. We then performed spatially resolved micro-photoluminescence *μ*PL at RT and at similar locations where the CL images had been taken, to map SPSs in that region (Figure 1B). In this image, the first features to notice are similar bright lines as compared to the CL image, which cannot be attributed to the same luminescence of SF observed at low-T in the UV. Indeed, luminescence of SF disappears above 100 K and, on top of that, the *μ*PL measurements were performed by exciting and collecting in the IR, while the CL is performed by collecting in the UV-visible. These bright lines might instead correspond to defects near the SFs, introducing deep levels within the bandgap. By performing spectrally resolved and second-order autocorrelation measurements at different spots, we could confirm the nonclassical nature of the emission of some of these bright spots. We studied four different locations and correlated the position of the SFs from the CL UV image with that of the SPSs from the IR *μ*PL image. Within the spatial resolution given by the IR *μ*PL, the bright spots systematically superimposed with a bright line of the low-T CL measurements, proving that all SPSs were located at the vicinity of a SF. This supports the hypothesis that in this sample, SFs, or the defects associated to them, play a role in the generation of SPSs. However, one should notice that not all SFs had SPSs nearby.

To test further the hypothesis, we grew a series of polar GaN on sapphire samples, which are known to be SF-less, under different growth conditions and performed again *μ*PL in the IR. As shown in Figure 1C, despite the absence of SF, we can still observe some bright spots with sharp spectral features and second-order autocorrelation functions at zero delay-times below 0.5 (highlighted by white circles), as discussed in the last section of the article. Even if all emitters are not necessarily related to the same defect, this proves that stacking faults are not a mandatory element to generate (at least) one type of such SPS emitting in the IR.

In the following, we studied the design and fabrication of photonic structures around these SPSs, which explains why we focused on polar GaN samples, as they benefit from a more advanced processing technology compared to their semi-polar counterpart [26]. More specifically, we worked with a 1 μm GaN on sapphire sample.

## 3 Fabrication of photonic structures

Different strategies exist for embedding SPSs into the maximum of the electric field intensity within a given photonic structure. In principle, if a way to fabricate deterministically the quantum emitters existed, then it would be possible to process the photonic structures randomly on the sample and position afterward the emitters at the correct locations [27–29]. In most cases though, the opposite scheme is used, where the cavity is processed around already identified emitters [6, 30]. Here again, several strategies coexist, with for example *in situ* lithography techniques where the localization and the writing (optical or electronical) are performed in the same chamber [31, 32]. Alternatively, one can use alignment marks, locate first the SPS with respect to the marks, and then fabricate the photonic structures at the predefined locations [33, 34]. In this article, we use this latter method to locate the SPS in the first place, by RT *μ*PL, and then perform the electron-beam lithography at the found location.

**Figure 2:**
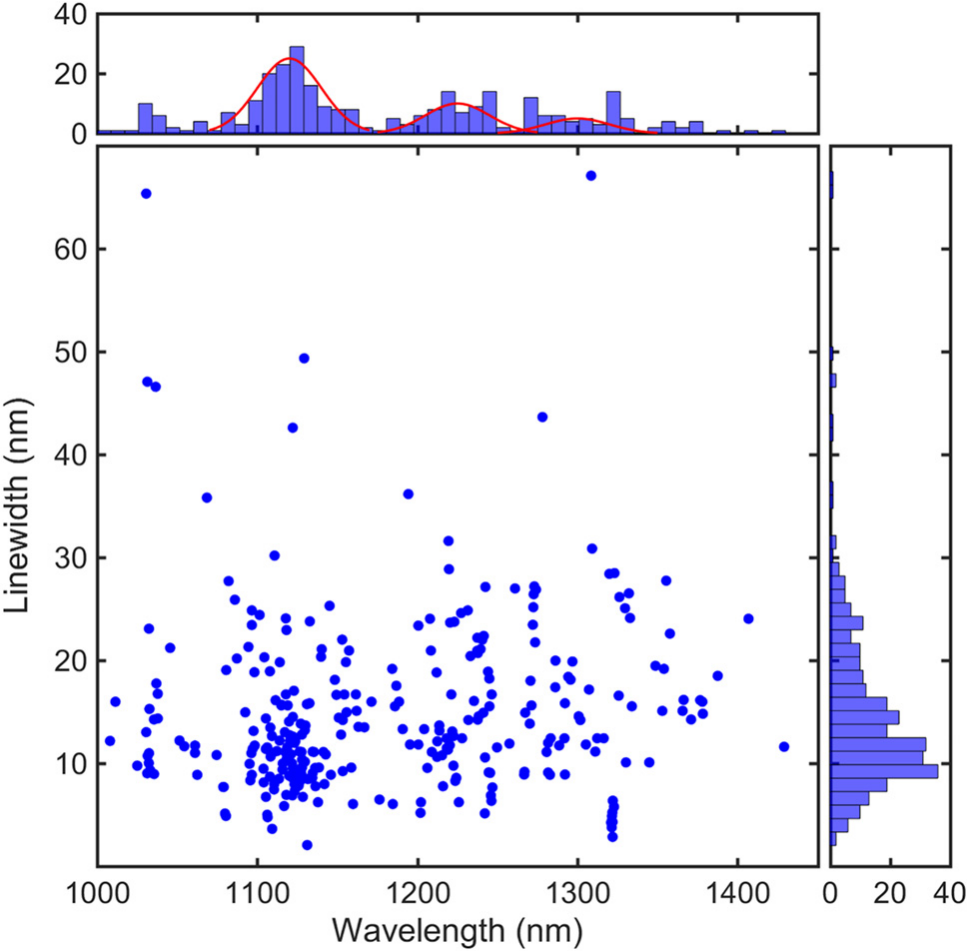
Distribution of room-temperature zero-phonon lines and linewidths of 300 telecom emitters observed in more than 30 different GaN samples and 2 AlGaN samples. The Gaussian distributions fitting the zero-phonon emission lines distribution are centered at 1120 nm, 1225 nm, and 1300 nm.

In total, we observed more than 300 emitters, on different samples, and display the distribution of their ZPL and linewidth on Figure 2. The corresponding histograms appear on top and on the side of the scatter plot. Some of these emitters were further characterized and used to build bullseye antennas around them.

We found ZPL distributed over a wider range than Zhou et al. [17] had observed, with emission wavelengths going from 1008 nm to 1428 nm. It is noteworthy that while the majority of linewidths concentrate around 12 nm, the wavelengths distribution (which can provide information on the nature of the defects) seems to peak around three central wavelengths 1120 nm, 1225 nm, and 1300 nm, which suggests the existence of, at least, three families of defects. Note that the telecom O band is fully covered by these emitters, showing their adequacy for fiber-based quantum communications.

To cope with the relatively large linewidth of these SPSs, we focused on low Q-factor structures, so as to maximize the spectral overlap between the SPS emission and the cavity resonance. We studied the coupling with a circular Bragg grating (CBG), also called bullseye (BE), for enhancing the out-of-plane extraction. BEs have been studied extensively for more than 30 years now, initially for enhancing emission and collection of distributed-feedback semiconductor lasers [35–37] and more recently for enhancing the emission and collection of quantum emitters [38]. Such structures have even already been demonstrated in GaN for quantum dots in the UV [39] but never in the telecom range.

A BE consists in a central circular cavity surrounded by a grating, which plays the roles (1) of an in-plane mirror enhancing the confinement in the cavity and (2) of an outcoupler to extract the emitted photons along the vertical direction. Variations of this basic geometry, mostly based on symmetry-breaking arguments, enable to add other functionalities through the engineering of the wavelength- and polarization-dependent resonances; this more sophisticated schemes can be useful for resonant excitation or for multiplexing [40, 41]. Furthermore, BEs are compatible with electrical contacts, which can enable charge noise mitigation [42], similarly to nanopillars [6].

### 3.1 Simulation of a BE spectral response

As our emitters each have different ZPL spread over a spectral band larger than 400 nm, we needed to find a methodology to optimize in general the geometric parameters of BE spanning a large wavelength range. The goal here was, therefore, to obtain “calibration curves” for the spectral response of BE structures optimized for different geometries. Once this “calibration ruler” would have been established, there would be no need to optimize individually the geometry for each wavelength. To establish it, we employed 3D finite-difference time-domain (FDTD) simulations. By simulating the time-response of a broadband pulse, we could switch to the frequency domain by means of Fourier transform.

As we have seen in Section 2, we only performed analysis considering a sample of 1 μm thick c-GaN on a sapphire substrate. Refractive indices of the GaN and of the sapphire were taken respectively from [43, 44].

A BE is usually seen more like an antenna than an actual cavity, but we choose here to optimize this second aspect. Indeed, the larger GaN/Air index contrast as compared to the GaN/Sapphire one would always foster a higher extraction efficiency toward the substrate. Thus, in the current stage of development, we preferred to see the grating surrounding the cavity mainly as a ring-shaped mirror rather than as an out-coupler. In future research, the SPS emission toward the sapphire substrate would need to be suppressed, either by using a bottom mirror (metallic or DBR) or making suspended structures, as currently done for GaN-on-Si photonic crystals [45, 46].

Thus, the aim was to obtain a resonant cavity surrounded by a fully etched (i.e., from the surface down to the GaN/Sapphire interface) Bragg mirror. For that, the thickness of each GaN and air rings should be an odd integer multiple of one fourth of the wavelength in the medium, according to the Bragg condition. Thinking in terms of pitch and duty cycle (DC), we get

$$d_{\text{GaN}}=pitch*DC=\frac{\lambda }{4n}$$

To optimize the cavity and BE design, we placed a dipole polarized along the *z*-axis at the center of the cavity, with the maximum of its emission being directed in-plane. The dipole was set to emit over a broad band from 500 nm to 2 μm. To maximize the interaction of this dipole with the cavity mode, it should be placed at an antinode of this latter. It obliged the diameter of that cavity to be an odd integer multiple of one-half of the wavelength. In addition, to cope with potential alignment uncertainties during the fabrication, we wanted to avoid having the smallest cavities. For these reasons, we set the cavity diameter as 5 times the half wavelength. Once again, in terms of pitch and DC, we should obtain:

$$r_{\text{cavity}}=5\;*\;pitch\;*\;DC=5\;*\;\frac{\lambda }{4n}$$

**Figure 3:**
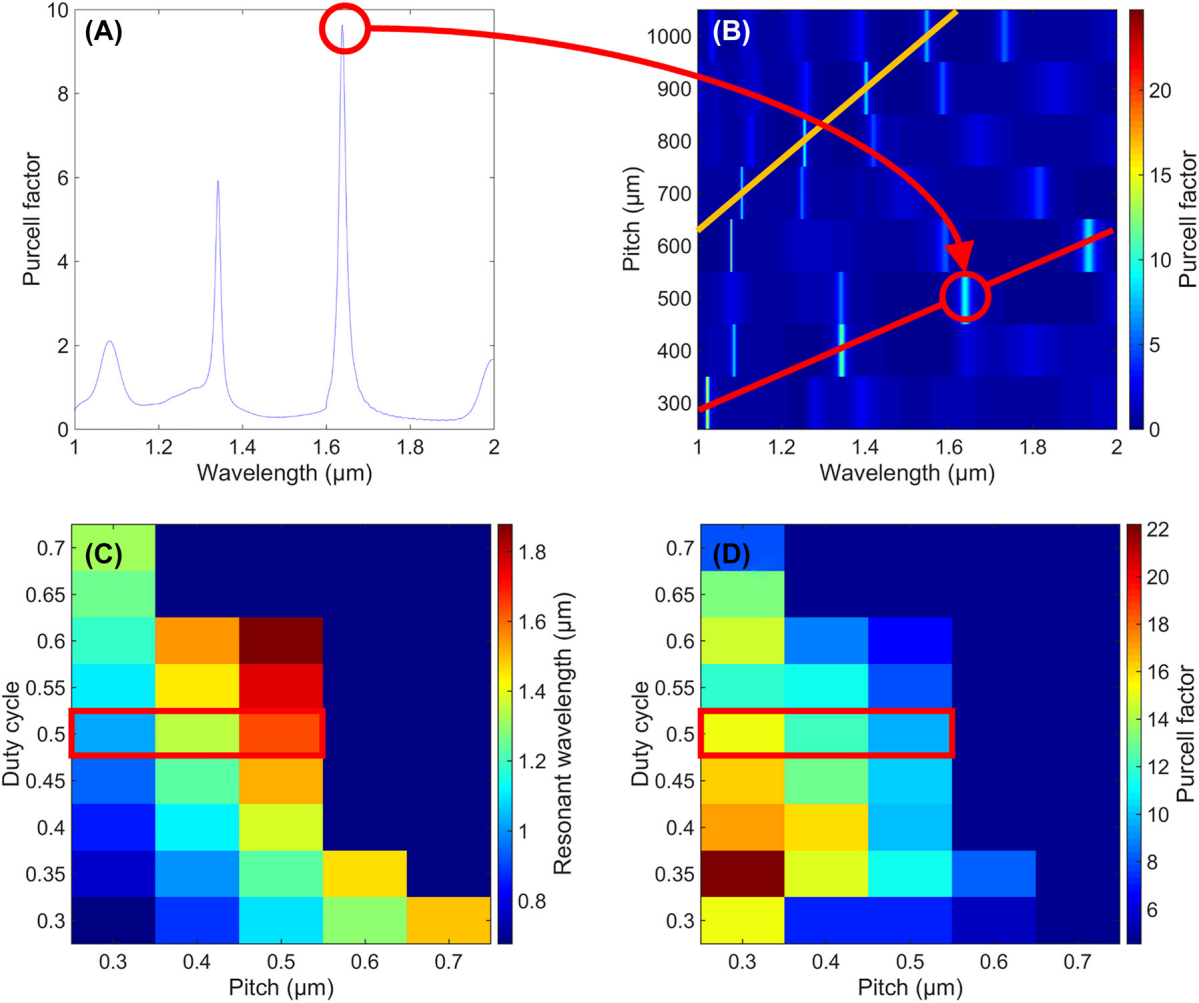
Simulations result for the Purcell factor: (A) as a function of the wavelength for one set of parameter (pitch, DC) = (500 nm, 0.5). The circle highlights one resonant order that we extract and compute thereafter. We call “first-order” the resonances corresponding to those highlighted in red. (B) As a function of the pitch and the wavelength. The DC is constant for the whole image at 0.5. The lines highlight again the resonances that we extract, containing the previous red points of subfigure (A), as well as a yellow line corresponding to the denominated “second-order” resonance mode. (C) and (D) Show the extracted resonant wavelength and corresponding Purcell factor, as a function of pitch and DC, for the denominated “first-order.” The red rectangles correspond to the same extracted values shown by the red line of subfigure (B).

As the height of the GaN layer was fixed by our sample to 1 μm, the only parameters left to be optimized were the pitch and DC of the surrounding grating (that is to say, the widths of the GaN and air vertical layers). To build up our calibration ruler, we swept them across different values and recovered for each pair (pitch, DC) a wavelength-dependent Purcell factor. We calculated it as the amount of light emitted by the dipole in the structure, divided by that in a homogeneous medium. An example is shown for (pitch, DC) = (500 nm, 0.5) on Figure 3A, where we extracted one order of resonance, which we arbitrarily labeled “first-order” (red circle). This resonance corresponds to the combination of one specific order of the central cavity mode combined with one specific order of the surrounding Bragg mirror. The evolution of such resonance can be visualized for example by displaying as a color plot, for one specific DC of 0.5 and a pitch varying along the *y* axis on Figure 3B. Once again, the extracted “first-order” resonance of Figure 3A and its evolution as the pitch changes is displayed in red. We also extracted another order of resonance, displayed in yellow, and arbitrarily labeled “second-order.”

We repeated that process of extracting such resonances for each combination of geometric dimensions. Figure 3C and D show, respectively, the wavelength and the associated Purcell factor of the labeled “first-order” resonance, as a function of the pitch and the duty cycle. The pixels framed in red correspond to the red line of Figure 3B.

**Figure 4:**
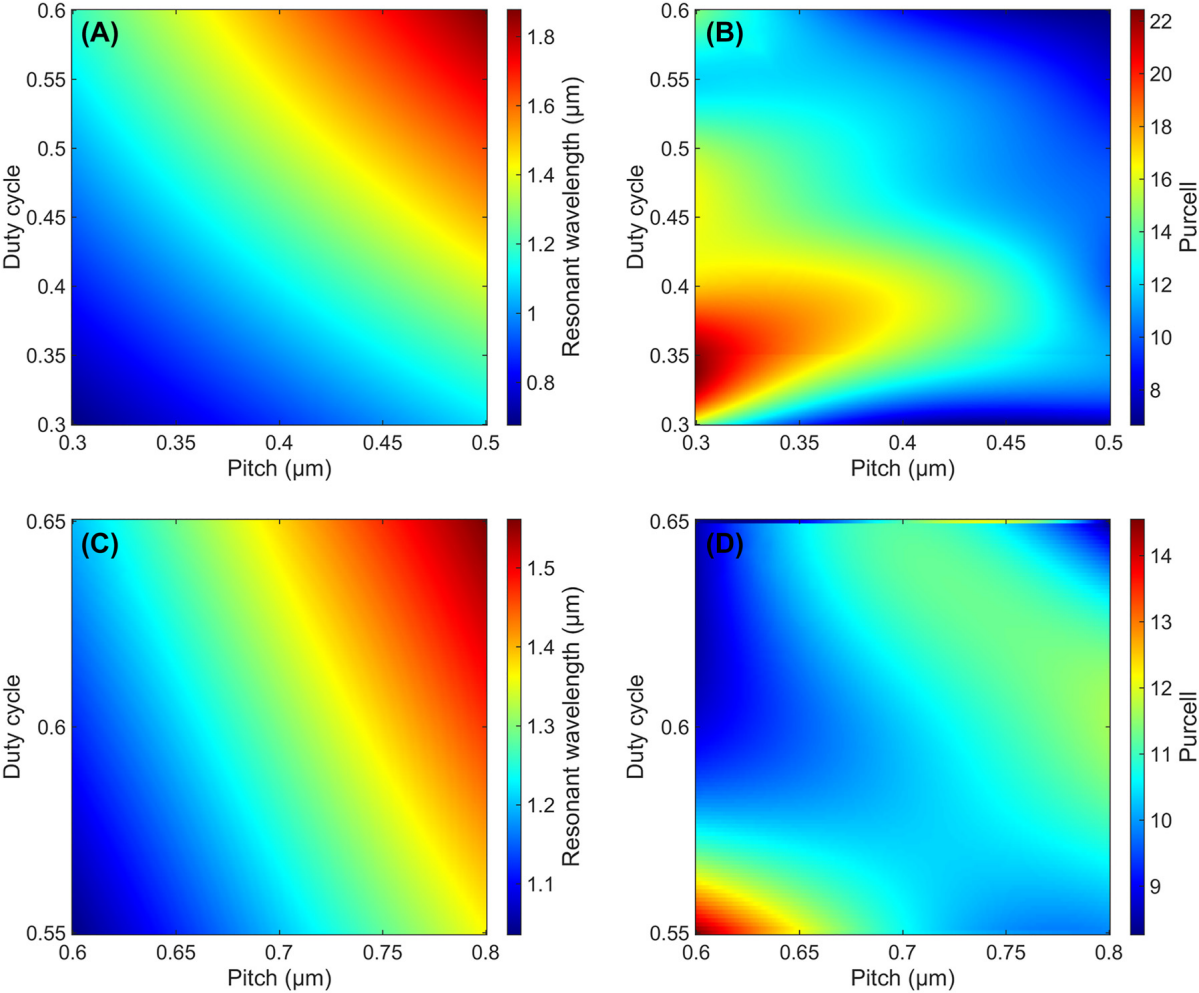
Interpolated color plots of the resonant wavelength (left: A, C) and associated Purcell factor (right: B, D), for two different resonance orders that we have extracted: for the denominated “first-order” (top: A, B) and “second-order” (bottom: C, D).

We then performed spline interpolation, both horizontally and vertically, to obtain the total evolution of each *n*th order resonant mode as a function of the dimensions of the grating. Figure 4 shows a final example of such interpolated matrix, (A) and (B) for the labeled “first-order” resonance (highlighted in red Figure 3) and (C) and (D) for the “second-order” resonance (highlighted in yellow Figure 3).

In the following, we used these “calibration curves” to design the BE at any wavelength, without the need to perform further simulation optimization. Of course, one would need to re-build such curve for any change in one single parameter that we considered constant such as the material, or the etch-depth, the thickness of the GaN layer, etc.

### 3.2 Processing of the BE

A first issue to take into consideration for the fabrication was the need to deterministically process the structures. Indeed, the low density of SPS combined to the resonant spectral width of our cavities (some nanometers), which is much smaller than the spectral emission range of our SPS (hundreds of nanometers), prevented us from processing cavities randomly over the GaN surface: the probability to have an emitter spectrally and spatially matched with the cavity would be extremely low in our case.

To overcome this, we proceeded with alignment marks made by optical lithography, in the shape of a matrix of small 6 by 8 μm^2^ rectangles, spaced by 100 μm. We thus found the emitters with respect to the marks and processed the structures on top, using the corners of the nearest rectangle as alignment reference. Such marks were not optimal for perfect alignment but were sufficient to achieve the demonstrations of this article. In order to see the marks under the e-beam resist, we fabricated them with titanium and gold, 10 and 70 nm thick, respectively.

Regarding the etching, it is important to note that, compared to other materials, III-N materials are in general chemically stable, with relatively large energy bond in case of Ga-N, which leads to the need for specific dry etching recipes to obtain vertical and smooth profiles [47]. In this work, we etched with a chlorine-based plasma in an inductively coupled plasma (ICP) reactive-ion etching (RIE) chamber and, thus, we used a metallic Nickel mask 60 nm thick, with a GaN/metal selectivity above 10. This enabled us to fully etch the GaN [48].

To successfully lift-off the BE, and prevent the random removal of rings, we spincoated PMMA 495 K A6 with a thickness that is 6 times that of the metal. We thus deposited 360 nm of resist and then soft baked at 130 °C for 2 min. The electron beam lithography (EBL) was done with a scanning electron microscope (SEM) Supra 40 by Zeiss equipped with a Raith Elphy System. We set the acceleration voltage at 30 kV and the beam current around 15 nA.

To secure the lift-off with that thickness of resist, we applied an overall dose of 350 μC/cm^2^. However, with such values, the developed areas would be much larger than the written one. Thus, we also applied a dimension correction coefficient on some features of the structures, that is, we diminished the ordered dimension of the EBL writing, to get in the end the desired one. The central cavity was naturally overexposed, leading to an overexposed first ring too, which might cause the merging of both features. The width of the next rings was sufficiently identical to apply on them the same coefficient, except for the very last ring. However, since the number of rings was sufficiently large, in this case 20, the influence of the outermost one on the properties of the BE could be considered negligible. We thus had 3 coefficients to find: for the central cavity, for the first ring, and for all the other rings. These dimension correction coefficients needed to be found experimentally for each set of dimension of BE, by sweeping them and measuring. They were also greatly influenced by all the parameters of the process, in particular the resist thickness and the writing dose.

**Figure 5:**
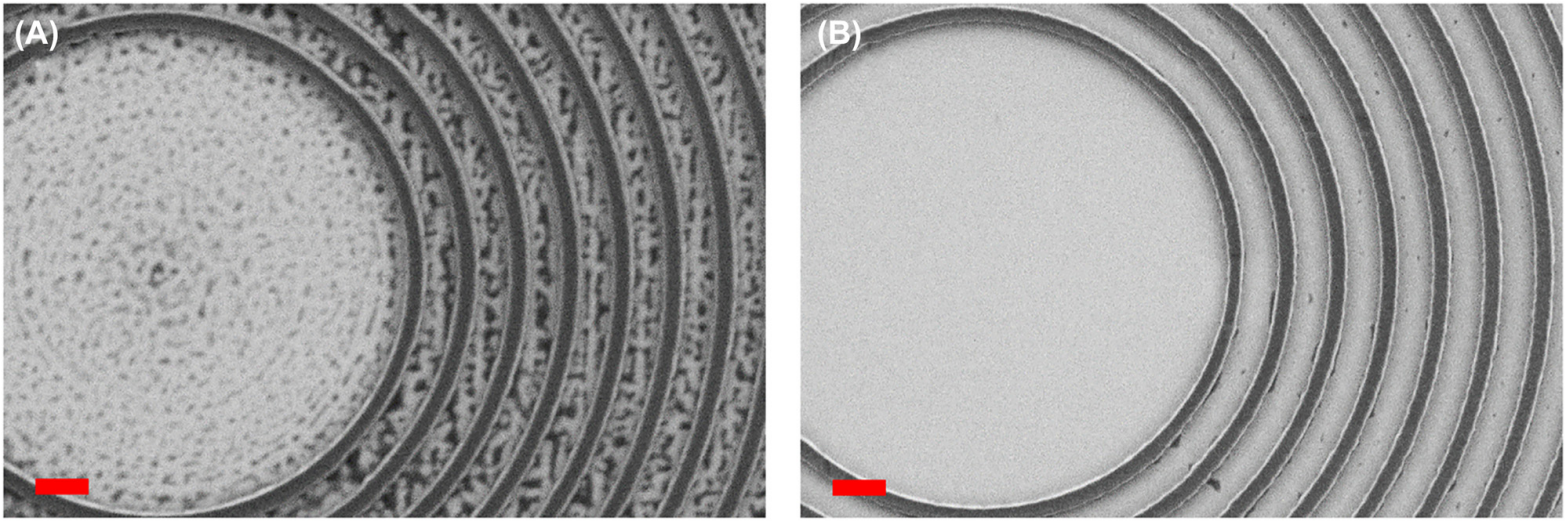
SEM images of the same BE, right after development (A) and after additional 10 s O_2_ plasma (B). Dark areas correspond to resist, and white areas correspond to the GaN below. Scale bars represent 1 μm.

After writing, we developed statically into a 1:3 MIBK:IPA solution for 1 min 30 s and stopped the development with 30 s of IPA. We dried carefully with an N2 gun. By observing under SEM at this stage of the process, we found out that some residues of resist could remain in regions that should be fully exposed, as shown in Figure 5A. In order to solve the latter issue, we “flash-etched” the PMMA with a 10 s O_2_ plasma in an electron cyclotron resonance (ECR) RIE Oxford System 100. Parameters of the treatment are as follows: 50 sccm O_2_, 20 mTorr chamber, 20 W RF bias power, and 200 W ECR power. We measured that 20 nm of resist are etched with that recipe, by measuring the thickness of the resist before and after with a surface profilometer Veeco Dektak 8. Figure 5B shows the same CBG as figure A, but after such a treatment. The exposed areas were indeed much cleaner but the thickness of the resist walls also a bit smaller due to side attack of the not-perfectly anisotropic plasma. This was compensated by adapting the dimension coefficients mentioned above.

Metal has deposited with an electron-beam evaporator Alliance Concept EVA 450. The deposition was made at a rate of 0.5 nm/s. We found out that the vacuum level of the deposition chamber had few impact on the next steps.

For the lift-off, we tried two different solvents (acetone and 80° Remover PG) and different methods (long static lift-off, magnetic rotater, ultrasonic bath). Thanks to the thick resist, this process supports a relatively fast lift-off: 20 min of acetone in ultrasonic bath were enough to perfectly remove all residues.

To etch the structures, we used a 210IL Corial ICP-RIE based on 15Cl_2_/2CH_4_/2Ar, with a RF bias power of 15 W and an inductive ICP power of 600 W. Pressure and temperature are, respectively, 3 mTorr and 20 °C. This recipe was good to etch relatively thin layers of GaN, but would lose verticality and smoothness after a certain thickness, as the Nickel mask would start to be etched on the side. Finally, the Nickel was simply removed by 10 min of piranha H_2_SO_4_ : H_2_O_2_ with a 3:1 ratio.

**Figure 6:**
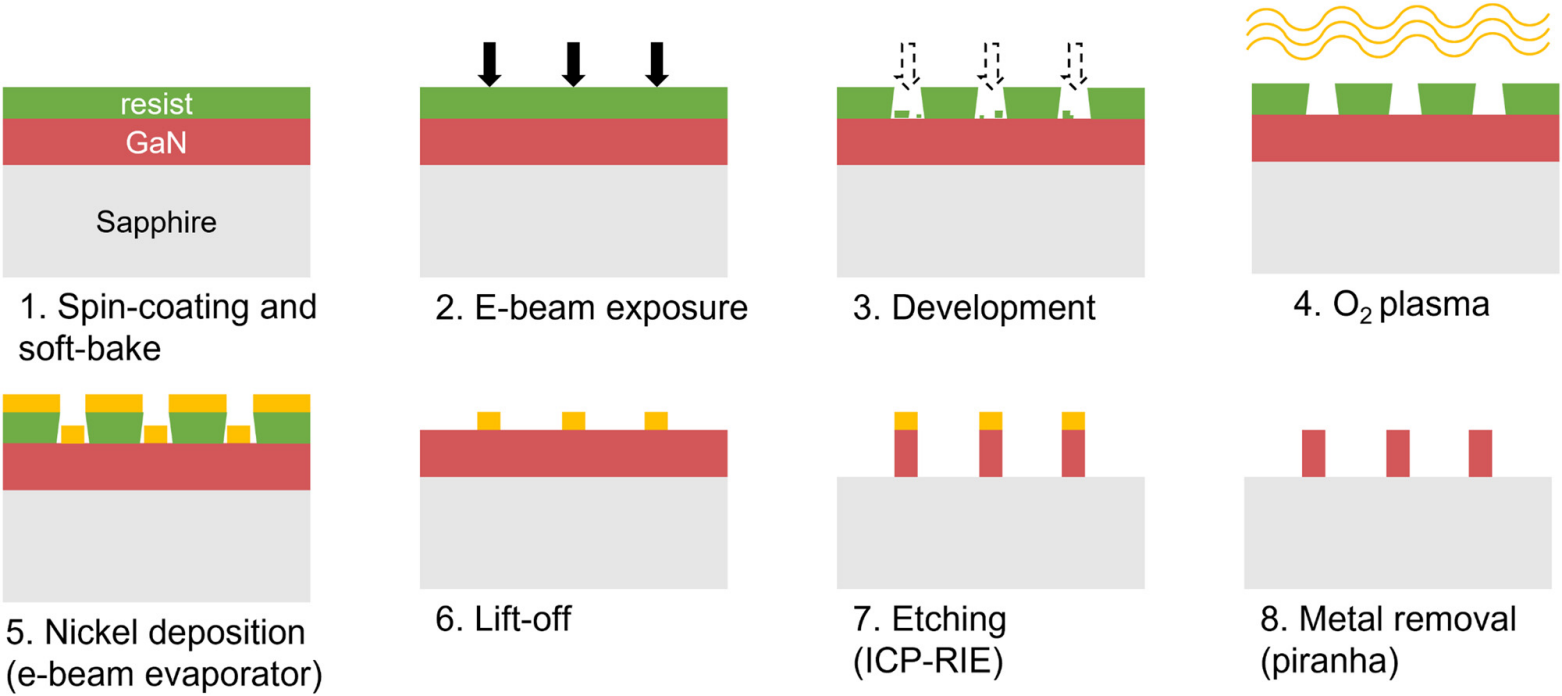
Schematic of the final process used for the fabrication. Precise description of each step can be found in the main text.

The overall process, summarized in Figure 6, showed both strong robustness and easy implementation. However, due to the relatively high thickness of resist, we had to give up on producing “first-order” resonance bullseye structures: indeed, thicknesses of PMMA larger than 300 nm will complicate the fabrication of features smaller than 200 nm, which are required for these so-called “first-order” BE. Thus, for coupling with the quantum emitters that will be discussed next, we employed the second-order sets of dimensions (see Figure 4C and D), which have pitch around 600 nm and DC around 0.6.

## 4 Single-photon emitters in the telecom coupled to BE antennas

In this last section, we will examine the emission characteristics of the telecom GaN single-photon emitters after processing the BE photonic structures around them. We fabricated five BEs around prelocalized emitters luminescing between 1150 nm and 1250 nm and found that four of them survived the processing while one was completely quenched after the photonic structure was fabricated. Several hypotheses could explain this quenching: the emitters, while luminescing at similar wavelengths, might arise from different defects with differing resilience to the fabrication processes; alternatively, the emitters could lie at different depths, thus those lying closer to the surface of the GaN thin film might be more sensitive to the etching step. Unfortunately, with the current statistic, it is not possible to conclude on the conditions for a GaN SPS in the telecom to survive the clean-room processing.

**Figure 7:**
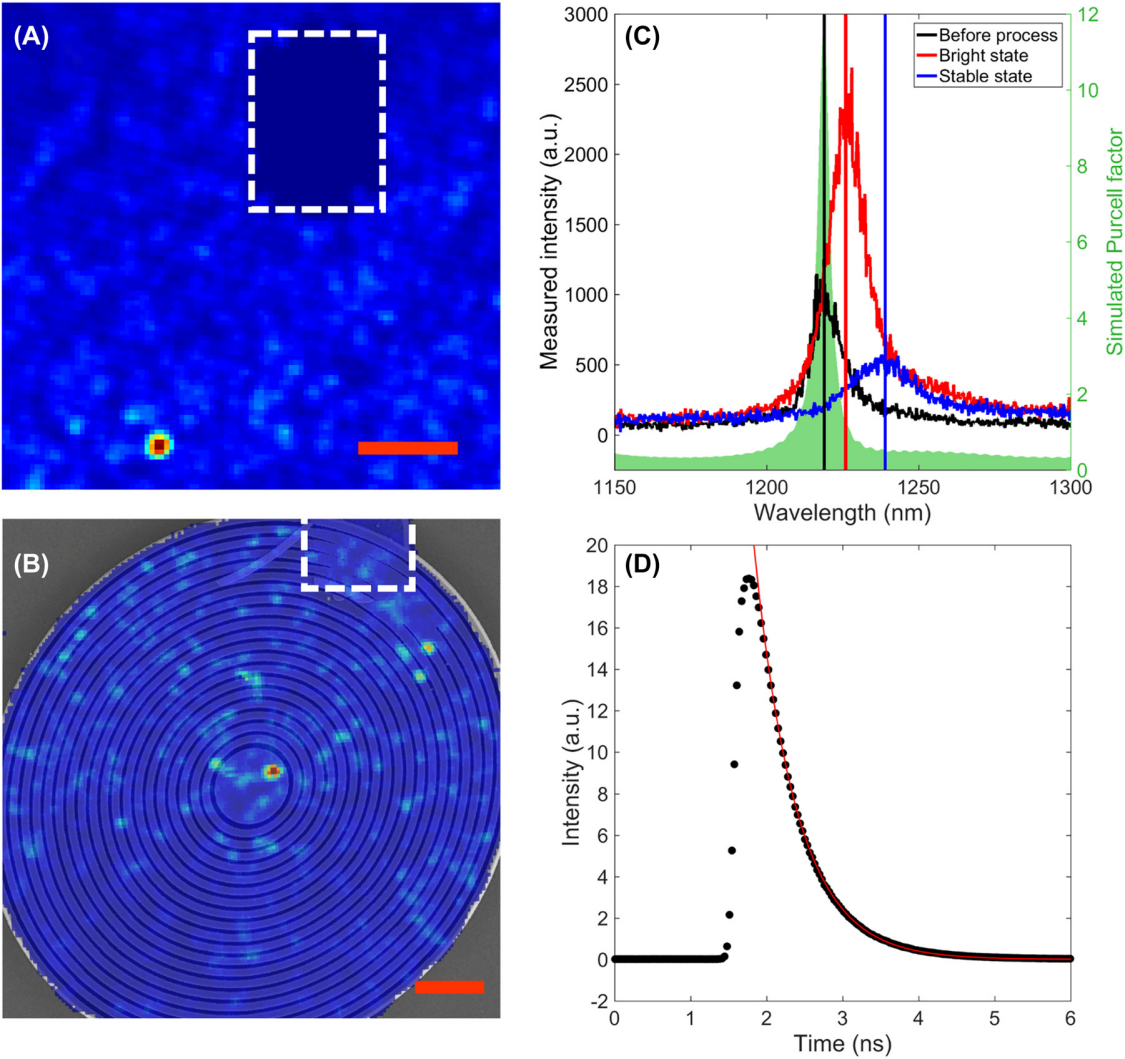
Bullseye antenna fabrication around GaN telecom single-photon emitters and optical characterization: (A) Spatially resolved room-temperature micro-photoluminescence of emitter E1, taken with a 100 nm bandwidth filter, before processing the bullseye antenna. (B) Scanning electron microscopy image of the bullseye antenna fabricated around emitter E1 superimposed on the spatially resolved room-temperature micro-photoluminescence of emitter E1, taken with a 100 nm bandwidth filter. The red scale bar represents 5 μm in (A) and (B). (C) Micro-photoluminescence spectra of emitter E1 before the fabrication process (black), after the fabrication process on the blinking high-intensity state (red), and after the fabrication process once the emission has stabilized on the low-intensity state (blue). The shadowed curve corresponds to the calculated Purcell factor of an emitter placed at the maximum electric field in a bullseye structure whose resonant wavelength corresponds to the emitter wavelength before process. (D) Time-resolved emission of emitter E1 once stabilized in the low-intensity state.

*μ*PL maps around an emitter (referred to in the following as E1) and the closest alignment mark before and after processing a BE structure are shown in Figure 7A and B, respectively. In Figure 7B, the *μ*PL map is superimposed over the corresponding SEM image of the photonic structure, with the SPS appearing as a bright spot in the central cavity of the BE. This proves that GaN-based infrared SPS can indeed survive the fabrication process and that neither the nonradiative surface states created upon etching [49] nor the strong strain-relaxation induced by the creation of free surfaces [50], quench the emission of these deep defects.

One of the objectives of embedding the SPS into photonic structures was to reduce its radiative lifetime through the modification of its electromagnetic environment, the so-called Purcell effect [51]. To maximize the Purcell factor, the emitter and the photonic mode must be both spatially and spectrally matched. As observed in Figure 7B, the spatial match is strongly compromised due to the current alignment procedure that we have employed, in particular due to the alignment marks themselves. This leads to a misalignment of about ∼1.5 μm (i.e., ∼2.5 *λ*_BE_/*n*_GaN_) in this example. Now that we have proven that GaN SPS sustain the fabrication photonic structures, alternative strategies will need to be tested to improve the spatial alignment, as discussed in Section 3.

**Figure 8:**
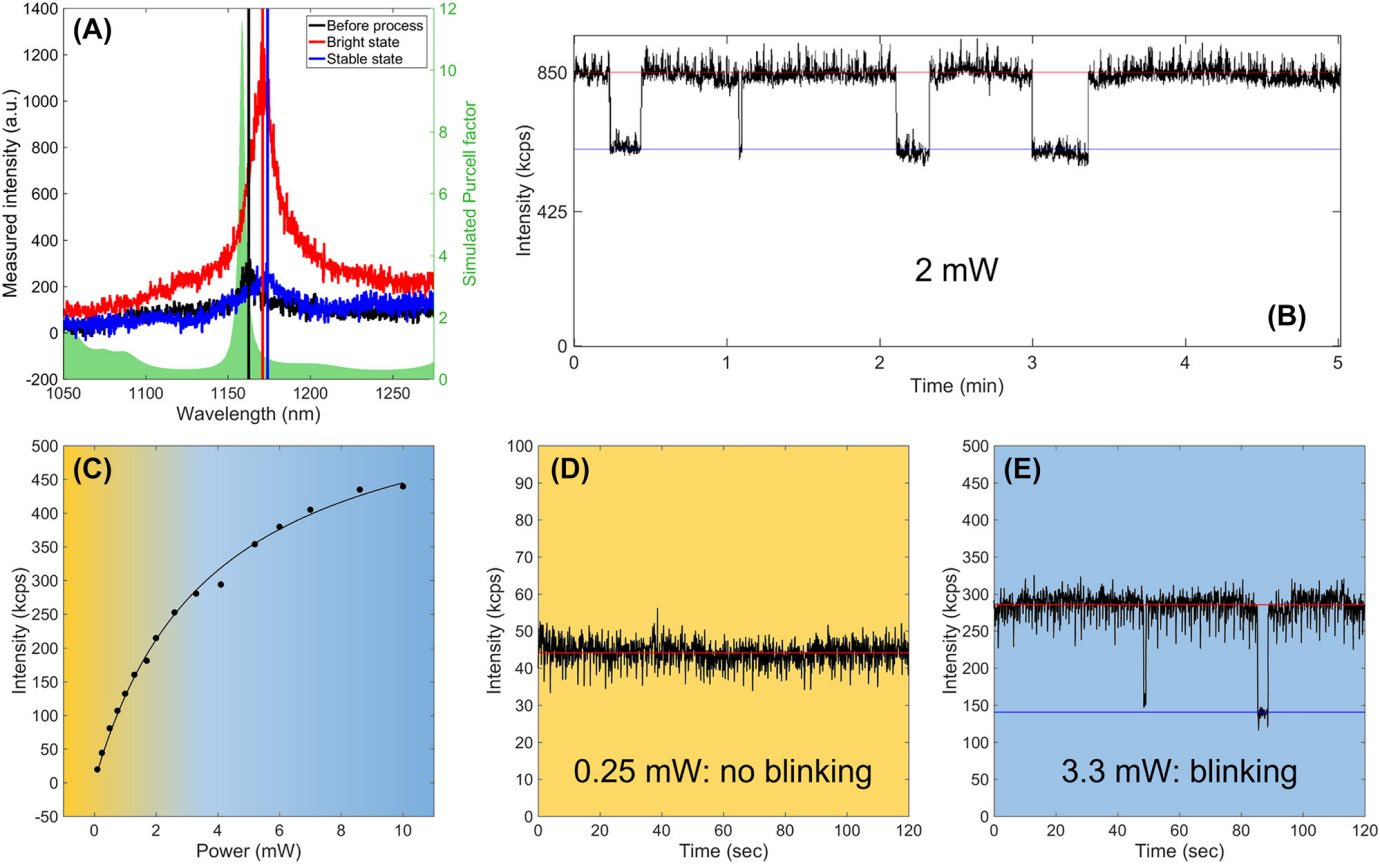
Optical characterization of telecom GaN single-photon emitters: (A) Micro-photoluminescence spectra of emitter E2 before the fabrication process (black), after the fabrication process on the blinking high-intensity state (red), and after the fabrication process once the emission has stabilized on the low-intensity state (blue). The shadowed curve corresponds to the calculated Purcell factor of an emitter placed at the maximum electric field in a bullseye structure whose resonant wavelength corresponds to the emitter wavelength before process. (B) Normalized intensity of the emitter E2 as a function of time illustrating the blinking (between the high- and low-intensity state). (C) Count rate of emitter E2 as a function of pumping power once stabilized in the low-intensity state. (D) and (E) Intensity of emitter E2 as a function of time once stabilized in the low-intensity state for pumping powers below (D) and above (E) the saturation pumping power.

Besides, Figure 7C shows that upon the fabrication of the photonic structure, the emission displays a wavelength shift, which is not surprising given the sensitivity of GaN to strain [50] and which is observed in all four emitters embedded into a BE. However, if strain relaxation was the only cause of this wavelength shift, we would have expected it to display systematically the same sign, which is not the case. Indeed, two emitters (E1 in Figure 7 and E2 in Figure 8) showed a redshift while the other two showed a blueshift. Thus, additional mechanisms, such as reconfiguration of the SPS defects structure, might come into play. Overall, given the spatial- and spectral-mismatch between the emitter and the photonic structure, we cannot expect a strong modification of the emitter’s lifetime, which we measured to be (after BE processing) in the order of 670 ps (Figure 7D). This lifetime is comparable to the one measured in GaN SPS emitting in the same wavelength range by Zhou et al. [17], pointing toward a negligible Purcell effect. Still, emitters E1 (analyzed in Figure 7) and E2 (analyzed in Figure 8) showed a 2.5 and 5 times intensity enhancement after processing the BE around them, suggesting an improvement of the extraction efficiency. Indeed, the red curves (“bright” state) in Figure 7C and in Figure 8A show the spectra measured on E1 and E2 under 2 mW laser excitation after processing the BE around them, while the black curves correspond to the same emitters measured under the same pumping conditions but before any processing. For completeness, we note that the other two emitters did not show any significant emission intensity enhancement after the fabrication of the BE.

Once embedded into the BE, all four emitters displayed an emission accompanied by random intensity fluctuations, so-called fluorescence blinking. An example of time trace corresponding to emitter E2 is shown in Figure 8B, where jumps between a high-intensity state (bright state) and a low-intensity state (dark state) occur within timescales in the order of minutes. Eventually, after a continuous exposure of few hours to the excitation laser (typically several milliwatts), the emitters stabilized into a lower intensity state. We then measured again the spectrum of both emitters, E1 and E2, and displayed them as blue curves in Figures 7C and 8A, respectively. Indeed, the *μ*PL map in Figure 7B was recorded with the emitter in the low-intensity state, which is still brighter than the surrounding background PL of GaN. A spectral redshift is observed when going from the high-intensity to the low-intensity state, although the value of the wavelength shift is different in both cases. Note that the spectrum of the high-intensity state presents a clear asymmetry, with the longer-wavelength side showing a slight wing as compared to the shorter-wavelength side. Indeed, the bright state spectrum can be fitted using the sum of two Voigt functions, each Voigt function centered at the respective wavelengths of the high- and low-intensity states. This helps to attribute the change in PL intensity to a spectral-diffusion–like behavior, similar to that observed in colloidal quantum dots [52] or in color centers in diamond [53]. This behavior has been often ascribed to local charge fluctuations near the defect that can cause its ionization, either quenching the emission and/or shifting the wavelength. This could be potentially controlled by adding a gate excitation [54]. Once stabilized in the low-intensity state, we measured the emission intensity as a function of pumping power (*P*_exc_), as displayed in Figure 8C. The measurement was performed with a 40 nm spectral filtering around the peak emission and the mean count rate (i.e., the counts per second) was obtained by averaging the measured intensity for 2 minutes. We fitted the curve with a first-order power dependence:

(1) $$I=I_{\text{sat}}\times \frac{P_{\text{exc}}}{P_{\text{exc}}+P_{\text{sat}}}$$

with *I*_sat_ the saturation intensity of the emitter and *P*_sat_ the corresponding pumping-laser saturation power. We found for E1 and E2, respectively, a saturation power of 3.8 and 1.2 mW, and a saturation intensity of 0.6 MHz and 0.3 MHz, respectively. Furthermore, while in the low-intensity state, exciting emitter E2 below the saturation power did not induce blinking as shown in the intensity time trace at 0.5 mW (Figure 8D). However, if the excitation power exceeded the saturation power, the emitter would begin to blink again as shown in (Figure 8E) at 3.3 mW, corresponding to 2.75 times the saturation power of emitter E2. Such excitation-related blinking has been observed in many other SPS and it is usually not due to an intrinsic property of the defect itself but rather associated again to environmental factors [55].

**Figure 9:**
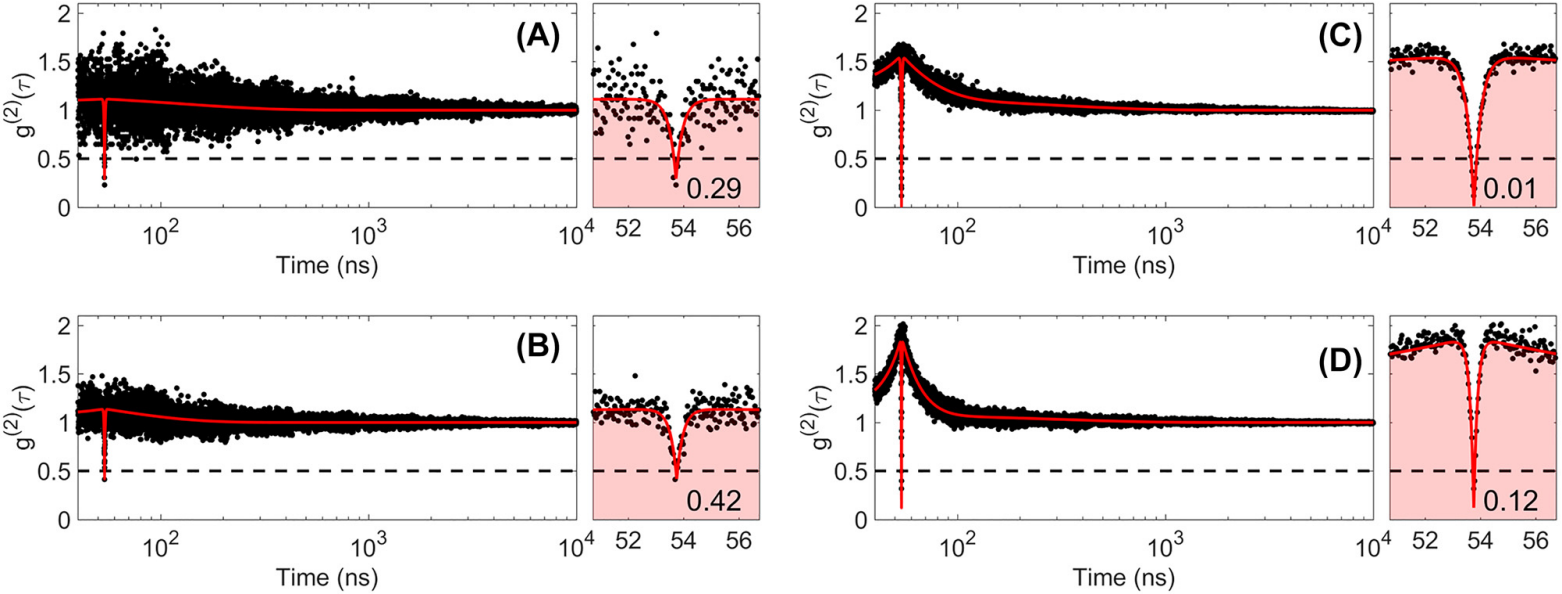
Measured second-order autocorrelation function *g* ^(2)^(*τ*) as a function of pumping power (0.5 mW, top; 1.5 mW, bottom) corresponding to an unprocessed emitter (left: A and B), and to an emitter embedded in a bullseye antenna (right: C and D, corresponding to emitter E1 in Figure 7). On the right of each long-delay measurements, zoom-in of the antibunching region. In red, fit of the second-order autocorrelation functions considering one (left: A and B) and two (right: C and D) shelving states.

To assess the purity of the singe-photon emitters and probe their time dynamics, we measured the second-order photon autocorrelation function (*g* ^(2)^(*τ*)) at room-temperature as a function of the pumping power for an unprocessed emitter (Figure 9A and B) as well as for emitter E1 once stabilized in the low-intensity state (Figure 9C and D). Interestingly, the two emitters showed *g* ^(2)^(0) values smaller than 0.5, attesting the quantum nature of both of them, for all the pumping powers, with the value at zero-delay time degrading as larger pumping powers are used. We fitted the *g* ^(2)^(*τ*) distributions with an empirical model for quantum emitters [56]:

(2) $$g^{\left(2\right)}\left(\tau \right)=1-C_{1}{\text{e}}^{-\tau /\gamma_{1}}+\sum C_{i}{\text{e}}^{-\tau /\gamma_{i}}$$

with *γ*_1_ the antibunching rate, *C*_1_ the antibunching amplitude, and *γ*_*i*_ the bunching rates and *C*_*i*_ their associated bunching amplitudes. Note that in this model, the number *n* of resolvable *i* timescales in the second-order autocorrelation measurement establishes a lower bound on the number of energy levels required to reproduce the curves on a rate-equation model [56]. Using this model, and without using any background correction, we obtain purity values for the emitter embedded in the BE photonic structure in the order of 99% for 0.5 mW pumping power, which corresponds to an emission rate of 0.08 MHz, and of 85% for 1.5 mW pumping power, which corresponds to an emission rate of 0.14 MHz. These large purities at room temperature, comparable to those of quantum emitters in hBN [56] and in lead-halide perovskite quantum dots [57], combined with large repetition rates, larger than those of the emitters just mentioned, attest of their technological potential for quantum communications applications.

It is also interesting to note that the two emitters shown in Figure 9, i.e., found in unprocessed and in processed samples, display photon bunching over timescales in the order of hundreds of nanoseconds, confirming the existence of other transition levels besides the radiative one at the origin of the single-photon emission. Even though a systematic study on several emitters would be necessary to extract statistically reliable conclusions, let us note that while the time dynamics of the unprocessed emitter can be well fitted using one single bunching rate (i.e., *i* = 1), the time dynamics of the emitter embedded in the BE requires the use of two bunching rates (i.e., *i* = 2), which indicates the existence of additional shelving states that might be generated during the fabrication of the photonic structures.

## 5 Conclusions

In this paper, we demonstrated the first embedment of room-temperature telecom single-photon emitters based on GaN into bullseye cavities. We described the growth of the material and our attempts to understand the origin of the defects, paying special attention to unraveling their possible connection with stacking faults, which were shown to be unnecessary. We then introduced our photonic structure design approach and the fabrication strategy to deal with the wide spectral distribution of emitters and their low density. Finally, we characterized the processed structures and compared the results with unprocessed GaN SPS.

While the processing of photonic structures around these randomly distributed emitters is possible, and the free surfaces do not annihilate the emission of the emitters, a permanent wavelength shift, followed by a jump into a low-intensity state, was observed. Meanwhile, even in this lower-intensity state, the single-photon emitters displayed purities in the order of 99% while keeping repetition rates in the order of hundreds of kHz. Thus, GaN telecom SPSs have a great potential for fiber-based quantum communications, but a deeper understanding of their origin and energy-level configuration, together with a more efficient coupling to photonic structures, seem necessary to exploit their full potential.

## 6 Experimental setup

The micro-photoluminescence (*μ*PL) setup consists of a confocal microscope equipped with 3D nanopositioner (Physik Instrumente, P-611.3S) to mount the sample for PL raster scan. Excitation and collection were performed through a single microscope objective (OLYMPUS, LCPLN 100× IR) with a NA of 0.85. The excitation is a CW laser diode emitting at 980 nm. The laser beam was filtered by a 1000 nm short pass filter (Thorlabs, FESH1000) to remove spectral signal below 1000 nm before entering the dichroic long pass beamsplitter (Thorlabs, DMLP1000R). The PL signal was collected via a single mode fiber into either the low-jitter IR superconducting single-photon detector (Scontel) for photon counting or the spectrometer with a InGaAs camera (ANDOR) for spectroscopy measurements. Spectra were taken at 2 mW excitation power. The second-order autocorrelation measurements were conducted with a HBT interferometer setup consisting of a 50:50 fiber beamsplitter before the superconducting single-photon detector. A time-correlated photon-counting card (Picoquant, Hydraharp) was used to record photon arrival events for two hours. The raw data were subsequently numerically processed to produce the second-order autocorrelation graphs. For the time-resolved measurements, we used 100 ps-long pulses with a repetition rate of 1 MHz.

The cathodoluminescence (CL) measurement was done with a Gatan MonoCL4 mounted on a FEB-SEM JEOL JSM700F. The dispersed light at the output of the monochromator was collected thanks to a CCD camera operating from 250 to 900 nm. Measurements were done at 77 K, with an acceleration voltage of 5 kV.

The GaN on PSS samples are the same one used in reference [25]. Instead, the polar GaN on sapphire samples were grown using a close-coupled showerhead CCS Thomas Swann MOCVD reactor equipped with a 3 × 2 inches susceptor. The used precursors were trimethylgallium (TMGa) and ammonia (NH3). The growth of the GaN was preceded by a nitridation of the sapphire, a SiN surface treatment, and a low-temperature GaN buffer.
